# A case of post-transplant adult T-cell leukemia/lymphoma presenting myelopathy similar to but distinct from human T-cell leukemia virus type I (HTLV- I)-associated myelopathy

**DOI:** 10.1186/2193-1801-3-581

**Published:** 2014-10-04

**Authors:** Toyotaka Kawamata, Nobuhiro Ohno, Kota Sato, Masayuki Kobayashi, Norihide Jo, Koichiro Yuji, Ryuji Tanosaki, Yoshihisa Yamano, Arinobu Tojo, Kaoru Uchimaru

**Affiliations:** Department of Hematology/Oncology, Research Hospital, The Institute of Medical Science, the University of Tokyo, 4-6-1 Shirokanedai, Minato-ku, Tokyo, 108-8639 Japan; Division of Molecular Therapy, Department of The advanced Clinical Research Center, The Institute of Medical Science, the University of Tokyo, 4-6-1 Shirokanedai, Minato-ku, Tokyo, 108-8639 Japan; Department of Blood Transfusion and Cellular Therapy, National Cancer Center Hospital, 5-1-1 Tsukiji, Chuo-ku, Tokyo, 104-0045 Japan; Department of Rare Diseases Research, Institute of Medical Science, St. Marianna University Graduate School of Medicine, Sugao, Miyamae-ku, Kawasaki, Kanagawa, 216-8512 Japan

**Keywords:** Adult T-cell leukemia/lymphoma, Post-transplant myelopathy, HTLV-I-associated myelopathy (HAM), Neopterin, CXCL10 (IP-10)

## Abstract

**Introduction:**

Adult T-cell leukemia/lymphoma (ATL) responds poorly to conventional chemotherapy, but allogeneic stem cell transplantation (allo-SCT) may improve disease prognosis. Herein, we report a female patient with human T-cell leukemia virus type I (HTLV-I)-associated myelopathy (HAM)-like myelopathy following allo-SCT for ATL.

**Case report:**

She developed crural paresis 14 months after allo-SCT. Initially, she was diagnosed with central nervous system (CNS) relapse of ATL and treated with intrathecal injection and whole brain and spine irradiation. Her symptoms recurred 5 months later, when a cerebrospinal fluid (CSF) specimen showed increased CD4 + CXCR3 + CCR4+ cell numbers and levels of neopterin and CXCL10 (IP-10).

**Discussion:**

These results suggest the possible involvement of a certain immunological mechanism such as HAM in her symptoms, irrespective of the lack of anti-HTLV-I antibody in her CSF. Because a definitive diagnosis of CNS manifestation of ATL is sometimes difficult, multi-modal laboratory data are required for differential diagnosis.

## Introduction

Human T-cell leukemia virus type I (HTLV-I) was the first retrovirus identified in humans, isolated from a patient with cutaneous lymphoma (Poiesz et al. 1980). HTLV-I is the cause of not only adult T-cell leukemia/lymphoma (ATL) (Uchiyama et al. 1977; Hinuma et al. 1981) but also HTLV-I-associated myelopathy (HAM)/tropical spastic paraparesis (TSP) (Osame et al. 1986), HTLV-I-associated uveitis (HU) (Ohba et al. 1989; Mochizuki et al. 1992) and infective dermatitis (McGill et al. 2012; de Oliveira et al. 2010).

ATL is one of the most intractable T-cell malignancies, and it responds poorly to conventional chemotherapy, with a median survival time (MST) of approximately 8 months (Shimoyama et al. 1988). Among such treatments, modified LSG-15 (mLSG-15) has shown the best results; in a previous study, the progression free survival (PFS) at 1 year among patients treated with mLSG-15 was 28% and the overall survival (OS) at 3 years was 24% (Tsukasaki et al. 2007). However, the improvement in survival time by mLSG-15 treatment is not satisfactory. Allo-HSCT is a promising treatment option to cure ATL because it may improve disease prognosis (Utsunomiya et al. 2001; Kami et al. 2003).

Herein, we describe a case of HAM-like myelopathy that was difficult to distinguish from central nervous system (CNS) relapse of ATL following allogeneic peripheral blood stem cell transplantation. This case report suggests that there might be immunological myelopathy after HSCT. In the present case, flow cytometric analysis of the cells in cerebrospinal fluid (CSF) was helpful to differentiate it from CNS relapse of ATL.

## Case report

A 63-year-old female patient recognized cervical lymph nodes swelling in October 2010. Lactate dehydrogenase (LDH) and serum corrected calcium levels kept within normal limit, but soluble interleukin-2 receptor (sIL-2R) elevated significantly at the initial visit (Table 1). Diagnostic imaging by computed tomography (CT) revealed systemic lymphadenopathies (cervical, axial, mediastinal, abdominal and mesenteric lymphadenopathy) before the following chemotherapy. Although appetite loss and abdominal distention were added with lymphadenopathy, any other abnormal finding of physical examination could not be detected. Her ECOG performance status was grade 1 before chemotherapy. She received cervical lymph node biopsy and pathological findings of cervical lymph node revealed T cell lymphoma compatible, and HTLV-I provirus DNA analysis (Southern blot) revealed monoclonal integration. Abnormal lymphocytes were not detected in peripheral blood (PB) and HTLV-I provirus DNA analysis of PB did not show monoclonal integration. She was diagnosed as ATL (lymphoma type). She has past histories of glaucoma and pulmonary cryptococcosis. None of ATL patient was in her family.

**Table 1:** **Laboratory data of onset of ATL (lymphoma type) in October 2010**

WBC	4100/μl	GOT	67 IU/L	CRP	0.06 mg/dl
Myelo	1.0%	GPT	72 IU/L	sIL-2R	5802 U/ml
St	8.0%	LDH	215 IU/L		
Seg	71.0%	ALP	277 IU/L	HTVL-I Ab	(+)
Ly	11.0%	γ-GTP	46 IU/L	HBs-Ag	(-)
Mo	8.0%	Alb	3.5 mg/dl	HBs-Ab	(-)
Baso	1.0%	BUN	15.6 mg/dl	HBc-Ab	(-)
RBC	423 × 10^4/^μl	Cre	0.58 mg/dl	HCV-Ab	(-)
Hb	13.2 g/dl	Na	142.4 mEq/L	HIV-Ab	(-)
Hct	39.0%	K	4.2 mEq/L	TPHA	(-)
MCV	92.2 fl	Cl	103.8 mEq/L		
MCH	31.2 pg	Corrected Ca	9.9 mg/dl		
MCHC	33.8%				
Plt	21.9 × 10^4^/μl				

**Figure 1:**
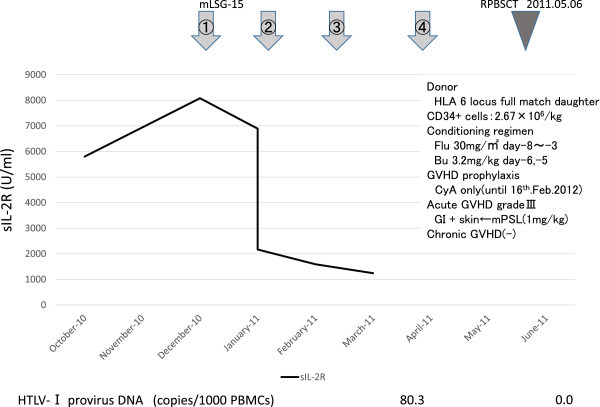
**Clinical course from conventional chemotherapy (mLSG-15) to allogeneic peripheral blood stem cell transplantation.** The patient received four sessions of mLSG-15 therapy and achieved complete remission (CR) before receiving allo-PBSCT.

She was referred to our hospital and received four sessions of mLSG-15 therapy in our hospital. Prophylactic intrathecal injection was performed twice, during chemotherapy and before allogeneic stem cell transplantation. No meningeal involvement of ATL cells was detected at that time. She went into complete remission (Response criteria for adult T cell leukemia-lymphoma from an international consensus meeting (Tsukasaki et al. 2009)) in April 2011. She received following allogeneic peripheral blood stem cell transplantation (allo-PBSCT) in the National Cancer Center Hospital (Tokyo, Japan) (Figure 1). The transplantation conditioning regimen consisted of fludarabine (30 mg/m^2^ per day for 5 days) plus busulfan (3.2 mg/kg per day for 2 days) and only cyclosporine A (CyA) was used for GVHD prophylaxis. Transplanted CD34-positive cells were 2.67 × 10^6^/kg and rapid engraftment was achieved. Grade III (gastrointestinal tract and skin) acute graft-versus-host disease (GVHD) was observed 1 month after transplantation, but it improved after treatment with methylpredonisolone (mPSL) (1 mg/kg). No chronic GVHD was observed. CyA was tapered gradually and discontinued 9 months after transplantation, in February 2012. After that point, only 5 mg/day predonisolone (PSL) was continued.

**Figure 2:**
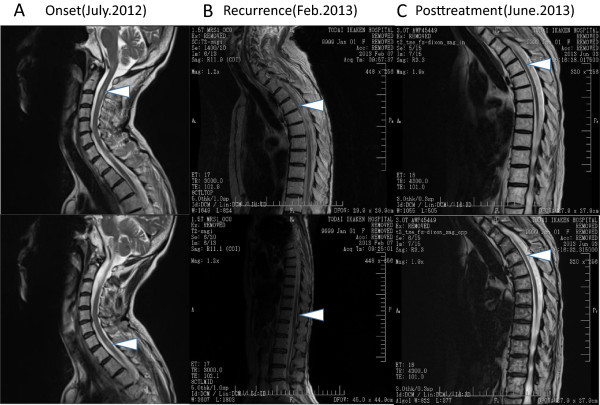
**MRI findings. A)** At the onset of neurogenic disorder in July 2012. Multiple high-intensity lesions in T2-weighted images (T2WI) of the medulla oblongata, cervical spinal cord, and thoracic spinal cord were revealed. **B)** Residual high-intensity lesion in Th3-7. **C)** Following treatment, the residual lesion in the thoracic spinal cord improved.

**Figure 3:**
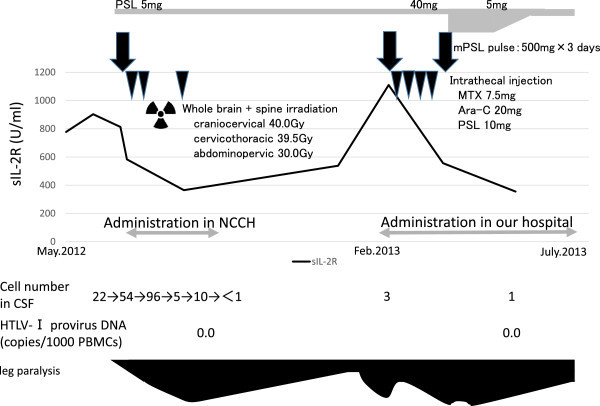
**Clinical course after onset of the neurogenic disorder.** The patient developed paraplegia 14 months after allo-PBSCT. Neurological findings were partially relieved following treatment with a high dose of mPSL accompanied by intrathecal injection of MTX + Ara-C + PSL and irradiation of the whole brain and spine. Three months later, her neurological deficit worsened again. Ultimately, her neurological disorder improved after treatment with a high dose of steroid.

In July 2012 (14 months after allo-PBSCT), the patient developed hemiparesis of the left side. Although left upper-limb paresis improved, lower-extremity paresis progressed to paraplegia. Magnetic resonance imaging (MRI) revealed multiple high-intensity lesions in T2-weighted images of the medulla oblongata, cervical spinal cord, and thoracic spinal cord (Figure 2A), and a CSF specimen showed increased cell counts (Figure 3). Morphologically, typical ATL cells such as flower cells were not detected in CSF, but abnormal small to middle size lymphocytes indistinguishable from ATL cells increased. She was diagnosed as CNS relapse of ATL, and received mPSL pulse, intrathecal injection of MTX 15 mg + Ara-C 40 mg + PSL 20 mg, and irradiation of the whole brain and spine. Following these treatments, the paraplegia improved gradually to such a degree that she could walk with a walker. During the course of these treatments, she was complicated by neurogenic bladder dysfunction, and diabetes insipidus.

**Figure 4:**
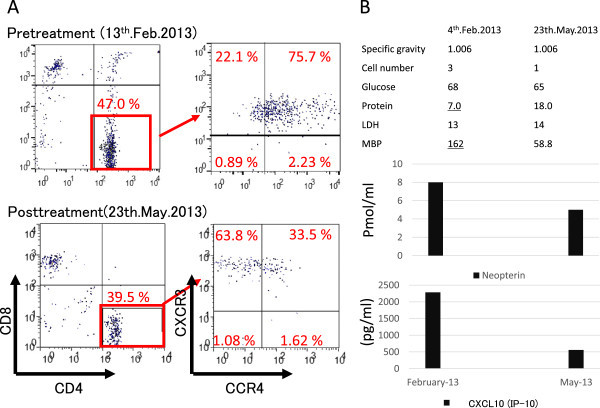
**CSF findings. A)** Flow cytometric analysis of CSF. Before treatment, the CD4 + CXCR3 + CCR4+ cell population was predominantly elevated. Following treatment, it decreased and the CD4 + CXCR3 + CCR4- cell population increased. **B)** Neopterin and CXCL10 (IP-10) concentrations in CSF. Before treatment, both neopterin and CXCL10 (IP-10) concentrations were significantly elevated. Following treatment, both biomarkers decreased to within the range of the therapeutic goal for HAM patients.

In January 2013 (20 months after allo-PBSCT), she again developed left lower-limb weakness, which gradually progressed. She was admitted to our hospital in February 2013. On admission, neurological examination revealed no abnormality of cranial nervous system, but abnormal reflex such as Babinski and Chaddock reflex in bilateral lower-limb. Thermal hypoalgesia under right Th4 and left Th6 dermatome was detected, but tactile sense was intact. She was accompanied with bladder dysfunction and severe constipation. Brain and spinal MRI revealed a residual spinal lesion at Th3-7 (Figure 2B). The cell numbers in CSF did not increase, but myelin basic protein (MBP) level was elevated (Figure 4B). Morphologically, ATL cells could not be detected in CSF. Flow cytometric analysis to determine the specific immunophenotype of CD4+ lymphocytes in CSF revealed an expansion of the CD4^+^CXCR3^+^CCR4^+^ cell population (Figure 4A), which conflicted with CNS relapse of ATL but was consistent with HAM (Natsumi et al. 2014). Furthermore, both the neopterin and CXCL10 (IP-10) concentrations in the CSF were significantly elevated (Figure 4B), although lower than those associated with aggressive HAM (14). Notably, the case was insufficient to fulfill the diagnostic criteria for HAM (Mitsuhiro 1990) because HTLV-I antibody (PA method) was negative in CSF.

Bacterial, fungal, and tuberculous encephalomyelopathies were excluded because no increase in cell numbers and no decline in glucose concentration in CSF were observed. Real-time polymerase chain reaction (PCR) testing for CMV, EBV, HSV, VZV, HHV-6, and JC virus in CSF showed negative results.

Serum soluble interleukin-2 receptor (sIL-2R) level was slightly elevated (Table 2), but significantly lower compared with that at the onset of ATL.

**Table 2:** **Laboratory data on admission to our hospital in January 2013**

WBC	4470/μl	GOT	15 IU/L	CRP	0.24 mg/dl
St	1.5%	GPT	33 IU/L	IgG	1390 mg/dl
Seg	64.0%	LDH	199 IU/L	IgA	51 mg/dl
Ly	14.0%	ALP	453 IU/L	IgM	352 mg/dl
Mo	19.5%	γ-GTP	87 IU/L		
Abnormal Ly	1.0%	TP	6.7 mg/dl	HTVL-I Ab	(+)
RBC	302 × 10^4^/μl	Alb	3.5 mg/dl	HBs-Ag	(-)
Hb	9.5 g/dl	BUN	9.8 mg/dl	HBs-Ab	(-)
Hct	29.4%	Cre	0.56 mg/dl	HBc-Ab	(-)
MCV	97.4 fl	Na	133 mEq/L	HCV-Ab	(-)
MCH	31.5 pg	K	4.0 mEq/L	HIV-Ab	(-)
MCHC	32.3%	Cl	96 mEq/L		
Plt	12.0 × 10^4^/μl	Corrected Ca	10.5 mg/dl		

Not all of the results necessarily corresponded to CNS relapse of ATL, although we could not exclude it. We treated her with mPSL pulse and intrathecal injection of MTX + Ara-C + PSL. After one course of mPSL pulse, her crural paresis improved dramatically to such a degree that she could pull up to standing after a few days. Although she was given intrathecal injections four times weekly, her crural paresis was gradually exacerbated and progressed to paralysis. mPSL pulse was performed again, but the effect was limited.

We examined her CSF again but there was no increase in cells, and ATL cells could not be detected by microscopic examination. Furthermore, the MRI findings improved over time (Figure 2C), although her neurological findings worsened and HTLV-I proviral DNA could not be detected repeatedly in peripheral mononuclear cells (PBMCs) after allo-PBSCT. No evidence of relapsed ATL could be found and we continued 5 mg/day PSL thereafter while she continued rehabilitation.

The results of CSF analysis in May 2013 showed the following improvements. In flow cytometric analysis, the CD4 + CXCR3 + CCR4+ cell population had decreased and the normal CD4 + CXCR3 + CCR4- cell population had increased. Both neopterin and CXCL10 (IP-10) had decreased to within the range of the therapeutic goal for HAM patients (Figure 4A,B). Her paralysis improved gradually and steadily only by rehabilitation, to such a degree that she could walk when holding onto parallel bars.

## Discussion

ATL with CNS involvement may occur during systemic progression of the disease and its frequency is estimated to be 10–25% (Kitajima et al. 2002). However, cases of CNS relapse without peripheral blood and lymph nodes of ATL have been reported (Marshall et al. 1998; Dungerwalla et al. 2005). In flow cytometric analysis of CSF of ATL patients, the CD4 + CXCR3-CCR4+ cell population is elevated. However, in the current case, the CSF fluid analysis revealed expansion of the CD4 + CXCR3 + CCR4+ cell population, which is consistent with HAM (Natsumi et al. 2014). Sato T et al. (Sato et al. 2013) reported increased neopterin and CXCL10 (IP-10) in HAM patients, and they were valuable biomarkers for disease progression of HAM. The neopterin and CXCL10 (IP-10) concentration in CSF paralleled the disease activity of HAM. The cut-off concentrations of neopterin and CXCL10 in HAM/TSP patients compared to HTLV-I infected non-HAM subjects are less than 5 pmol/mL and 200 pg/ml, respectively, and the CXCL10 (IP-10) concentration in the CSF of HAM patients with rapid progression is usually more than 5,000 pg/mL (Yamono, Y., personal communication). In the current case, we could not make a diagnosis of HAM because the CSF was negative for HTLV-I antibody in repeated examinations. Although the immunosuppressive status after allo-PBSCT might contribute, serum immunoglobulin levels were almost within normal limit at the same time period (Table 2) and there is not enough evidence to indicate false negative. In any inflammatory diseases of CNS, CXCR3+ cells but not CCR4+ cells were generally found in CSF(Misu et al. 2001). However, CXCR3 + CCR4+ double positive cells existed in her CSF. It was unlikely that CXCR3 + CCR4+ double positive cells emerged into CSF in nonspecific inflammatory condition. Given her background, we supposed these CCR4+ cells were HTLV-I infected cells, but the number of these cells was insufficient to measure HTLV-I viral load.

In the current case, neither CSF data nor clinical course consisted with CNS relapse of ATL. In case of ATL patients, CXCR3-CCR4+ T cell lymphocytes population expanded. Therapeutic effect was obtained from mPSL pulse rather than intrathecal injection. Furthermore, disease progression in the typical case of CNS relapse of ATL was more aggressive. We concluded some inflammatory condition caused by these HTLV-I infected cells may have developed HAM-like myelopathy.

CNS GVHD remains a controversial entity and it is difficult to establish an unequivocal diagnosis. Yet a few cases have been reported, who were suspected of CNS GVHD from brain biopsy or autopsy, their CSF showed predominant T-lymphocyte infiltration of donor origin (Kamble et al. 2007). In the current case, brain or spinal cord biopsy was not performed, and chimerism analysis of T cells in CSF was difficult because of the full-match HLA and sex-matched PBSCT. The number of T cells in CSF was insufficient to analyze chimerism using the short tandem repeat (STR) method. Neopterin (Niederwieser et al. 1984; Hempel et al. 1997) and CXCL10 (IP-10) (Mapara et al. 2006) levels in serum increase significantly in patients with active GHVD, but the levels in CSF are unknown. The possibility of active CNS GVHD could not be completely excluded. Both CXCR3 and CCR4 expression of T cells infiltrated in the CNS in patients with active CNS GVHD is unknown. It was no wander that CXCR3+ cells in CSF were found in nonspecific inflammatory condition such as CNS GVHD, but unlikely that CCR4+ cells were.

The patient’s neurological dysfunction seemed to fluctuate in parallel with the serum concentration of soluble interleukin-2 (sIL-2R) receptor (Figure 3). However, increased sIL-2R occurs not only with ATL relapse but also with HAM (Matsumoto et al. 1990), GVHD (Kami et al. 2000), and inflammatory neurogenic disorders caused by immunologic T-cell responses (Maier et al. 2009). Thus, it is difficult to make a definite diagnosis based on elevated sIL-2R alone.

In conclusion, we report a case with myelopathy without ATL relapse in the CNS. Flow cytometric analysis is helpful to differentiate immune-mediated encephalopathy or myelopathy from CNS relapse of ATL. If we encountered the patients suspected of CNS relapse of ATL, we should consider the possibility of inflammatory condition caused by HTLV-I infected cells. Further analysis of pathology are warranted.
